# Complete mitochondrial genome of banana new pest *Basilepta fulvipes* (Coleoptera: Eumolpinae) and phylogenetic analysis

**DOI:** 10.1080/23802359.2020.1797572

**Published:** 2020-07-25

**Authors:** Li-Na Liu, Si-Jun Zheng, Zhi-Xiang Guo, Xun-Dong Li, Jin-Bin Li, Li Zeng

**Affiliations:** a Agricultural Environment and Resources Institute, Yunnan Academy of Agricultural Sciences, Kunming, China; b Key Laboratory of Green Prevention and Control of Agricultural Transboundary Pests of Yunnan Province, Kunming, China; c Bioversity International, Kunming, China

**Keywords:** Mitogenome, *Basilepta fulvipes*, banana pest, phylogeny

## Abstract

*Basilepta fulvipes* (Motschulsky, 1860) is a banana new pest and mainly distributed in Eastern Asia. The complete mitogenome of *B. fulvipes* (GenBank accession number MT627597) is 15,762 bp in size, including 13 protein-coding genes, 22 transfer RNAs, 2 ribosomal RNAs genes and a noncoding D-loop region. The D-loop region is located between *12S rRNA* and *tRNA^Ile^*. The base composition of the whole *B. fulvipes* mitogenome is 41.66% for A, 8.89% for G, 34.32% for T and 15.12% for C, with a high AT bias of 75.98%. The present data could contribute to further detailed phylogeographic analysis and comprehensive control of this banaba new pest.

*Basilepta fulvipes* (Motschulsky, 1860) is a small beetle which belong to Eumolpinae, Chrysomelidae, Coleoptera. It is mainly distributed in Eastern Asia, while it is also a potential pest invaders to other regions (Roques et al. 2015). *B. fulvipes* has the ability to damage many crops and weeds, and recent years it was found as a banana new pest. *B. fulvipes* could damage the tender leaves and young fruit of banana, and form spots in leaves and fruit of banana, which seriously affect the quality and commercial value of banana fruit (Li et al. 2011). Elucidating the sequence and structure of *B. fulvipes* mitogenome is important for the diversity and phylogeographic analysis of this banaba pest, thus providing information for comprehensive control.

The specimen of *B. fulvipes* in present work was obtained from Baoshan, Yunnan, China (N 24°93′, E 98°89′), and deposited in the insect specimen room of Agricultural Environment and Resources Institute with an accession number AERI-G-20200312. Sequencing work of the complete mitogenome of *B. fulvipes* was performed by Illumina Nextseq500 in Beijing Microread Genetics Co., Ltd., with a total data volume 4 G (150 bp Reads). High-quality reads were assembled from scratch using IDBA-UD and SPAdes (Gurevich et al. 2013). Protein-coding genes (PCGs) of the *B. fulvipes* mitogenome were identified using BLAST search in NCBI, and tRNA genes were identified using the tRNAscan-SE search server (Schattner et al. 2005). The final assembled mitogenome was also verified on the MITOS web server (Bernt et al. 2013).

The *B. fulvipes* mitogenome is 15,762 bp in size (GenBank accession number MT627597), including 13 typical invertebrate PCGs, 22 transfer RNA genes, 2 ribosomal RNA genes and a noncoding control region (D-loop). The A + T content of the whole *B. fulvipes* mitogenome is 75.98%, showing an obvious AT mutation bias (Eyre-Walker, 1997). The D-loop region exhibits the highest A + T content (80.67%) in the *B. fulvipes* mitogenome. The gene order of *B. fulvipes* mitogenome is identical to other two Eumolpinae species previously reported (GenBank accession numbers JX412756 and KY039111)

Among all the 13 PCGs, 12 PCGs use standard ‘ATN’ as start codons, while *ND1* use ‘TTG’ as the start codon. As for stop codons, 8 PCGs had the common mitochondrial stop codon ‘TAA’, *ND4L* and *CYTB* terminated with stop codon ‘TAG’, while *COX2, COX3*, and *ND4* terminated with incomplete stop codon ‘T’. Similar cases could be found in other insect mitogenomes (Yin et al. 2012). It has been demonstrated that the incomplete stop codon ‘T’ could produce functional stop codon ‘TAA’ in polyadenylation processes by the addition of 3’A residues (Ojala et al. 1981).

All the tRNAs except *tRNA^Ser^ (UCU)* could be folded into the typical cloverleaf secondary structures. The unusual *tRNA^Ser^ (UCU)* has an incomplete dihydrouridine (DHU) arm. The 12S rRNA gene is located between *tRNA^Val^* and the D-loop region, while the 16S rRNA is located between *tRNA^Leu^* and *tRNA^Val^*. The locations of these two rRNA genes in *B. fulvipes* mitogenome are different to the ancestral insects mitogenome (Boore 1999).

Based on the concatenated 13 mitochondrial PCGs sequences of 17 species from Chrysomelidae, the neighbor-joining method was used to construct the phylogenetic relationship between *B. fulvipes* and 16 other Chrysomelidae insects (Figure 1). The phylogenetic analysis was performed by MEGA7 software (Kumar et al. 2016). Potential saturation of any PCG was assessed using DAMBE5 software (Xia 2013). *B. fulvipes* was firstly clustered with another Eumolpinae species (*Pseudocolaspis sp*.), and the phylogeny tree indicates that Eumolpinae has a close relationship with Cryptocephalinae. This mitogenome data might be also valuable for further phylogeography analyses in this banana new pest.

**Figure 1.**
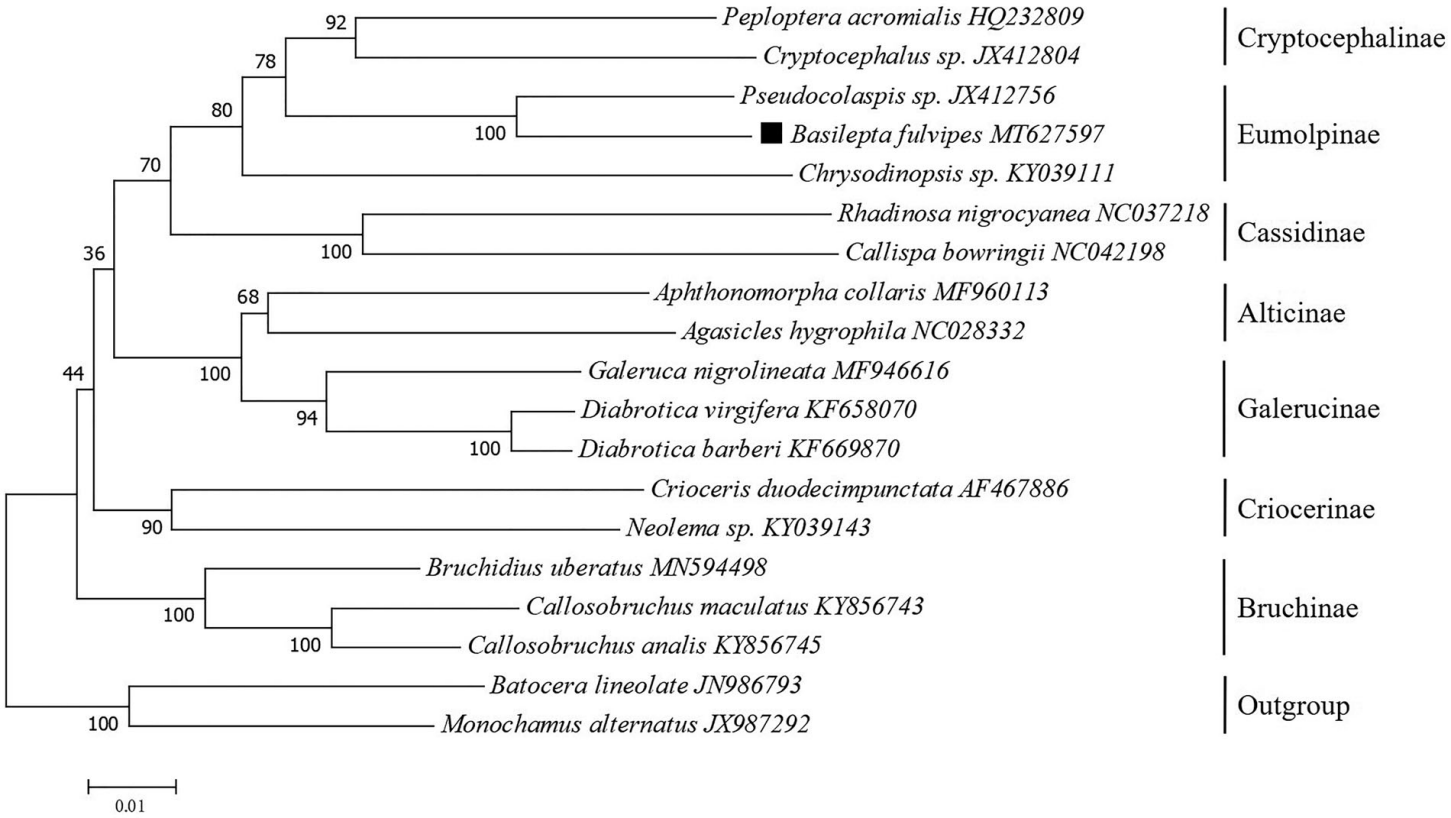
Phylogenetic tree showing the relationship between *B. fulvipes* and 16 other Chrysomelidae insects based on neighbor-joining method performed using 500 bootstrap replicates. *Batocera lineolate* and *Monochamus alternatus* were used as outgroup. GenBank accession numbers of each sequence were listed in the tree behind their corresponding species names.

## Data Availability

The data that support the findings of this study are openly available in “NCBI” at https://www.ncbi.nlm.nih.gov/, with a GenBank accession number MT627597.
